# Targeting CD73 with flavonoids inhibits cancer stem cells and increases lymphocyte infiltration in a triple-negative breast cancer mouse model

**DOI:** 10.3389/fimmu.2024.1366197

**Published:** 2024-03-27

**Authors:** Karan Mediratta, Sara El-Sahli, Marie Marotel, Muhammad Z. Awan, Melanie Kirkby, Ammar Salkini, Reem Kurdieh, Salman Abdisalam, Amit Shrestha, Chiara Di Censo, Andrew Sulaiman, Sarah McGarry, Jessie R. Lavoie, Zhen Liu, Seung-Hwan Lee, Xuguang Li, Giuseppe Sciumè, Vanessa M. D’Costa, Michele Ardolino, Lisheng Wang

**Affiliations:** ^1^Department of Biochemistry, Microbiology and Immunology, Faculty of Medicine, University of Ottawa, Ottawa, ON, Canada; ^2^Ottawa Institute of Systems Biology, University of Ottawa, Ottawa, ON, Canada; ^3^The Centre for Infection, Immunity, and Inflammation (CI3), University of Ottawa, Ottawa, ON, Canada; ^4^China-Canada Center of Research for Digestive Diseases (ccCRDD), Shanghai University of Traditional Chinese Medicine-University of Ottawa, Ottawa, ON, Canada; ^5^Ottawa Hospital Research Institute, Faculty of Medicine, University of Ottawa, Ottawa, ON, Canada; ^6^Department of Biotechnology, University of Azad Jammu and Kashmir, Muzaffarabad, Pakistan; ^7^Department of Molecular Medicine, Sapienza University of Rome, Rome, Italy; ^8^Department of Pathology, John Hopkins University School of Medicine, Baltimore, MD, United States; ^9^Centre for Oncology, Radiopharmaceuticals and Research, Biologic and Radiopharmaceutical Drugs Directorate, Health Products and Food Branch, Health Canada, Ottawa, ON, Canada; ^10^State Key Laboratory of Analytical Chemistry for Life Science, School of Chemistry and Chemical Engineering, Nanjing University, Nanjing, China; ^11^Centre for Biologics Evaluation, Biologics and Genetic Therapies Directorate, Health Canada, Sir Frederick G. Banting Research Centre, Ottawa, ON, Canada; ^12^Instituto Pasteur Italia – Fondazione Cenci Bolognetti, Roma, Italy; ^13^Ottawa Hospital Research Institute, Ottawa, ON, Canada

**Keywords:** triple-negative breast cancer, cancer immunotherapy, CD73, cancer stem cells, chemotherapy, drug repurposing, patient-derived xenograft, clinically translatable

## Abstract

**Introduction:**

Chemotherapy remains the mainstay treatment for triple-negative breast cancer (TNBC) due to the lack of specific targets. Given a modest response of immune checkpoint inhibitors in TNBC patients, improving immunotherapy is an urgent and crucial task in this field. CD73 has emerged as a novel immunotherapeutic target, given its elevated expression on tumor, stromal, and specific immune cells, and its established role in inhibiting anti-cancer immunity. CD73-generated adenosine suppresses immunity by attenuating tumor-infiltrating T- and NK-cell activation, while amplifying regulatory T cell activation. Chemotherapy often leads to increased CD73 expression and activity, further suppressing anti-tumor immunity. While debulking the tumor mass, chemotherapy also enriches heterogenous cancer stem cells (CSC), potentially leading to tumor relapse. Therefore, drugs targeting both CD73, and CSCs hold promise for enhancing chemotherapy efficacy, overcoming treatment resistance, and improving clinical outcomes. However, safe and effective inhibitors of CD73 have not been developed as of now.

**Methods:**

We used in silico docking to screen compounds that may be repurposed for inhibiting CD73. The efficacy of these compounds was investigated through flow cytometry, RT-qPCR, CD73 activity, cell viability, tumorsphere formation, and other in vitro functional assays. For assessment of clinical translatability, TNBC patient-derived xenograft organotypic cultures were utilized. We also employed the ovalbumin-expressing AT3 TNBC mouse model to evaluate tumor-specific lymphocyte responses.

**Results:**

We identified quercetin and luteolin, currently used as over-the-counter supplements, to have high in silico complementarity with CD73. When quercetin and luteolin were combined with the chemotherapeutic paclitaxel in a triple-drug regimen, we found an effective downregulation in paclitaxel-enhanced CD73 and CSC-promoting pathways YAP and Wnt. We found that CD73 expression was required for the maintenance of CD44^high^CD24^low^ CSCs, and co-targeting CD73, YAP, and Wnt effectively suppressed the growth of human TNBC cell lines and patient-derived xenograft organotypic cultures. Furthermore, triple-drug combination inhibited paclitaxel-enriched CSCs and simultaneously improved lymphocyte infiltration in syngeneic TNBC mouse tumors.

**Discussion:**

Conclusively, our findings elucidate the significance of CSCs in impairing anti-tumor immunity. The high efficacy of our triple-drug regimen in clinically relevant platforms not only underscores the importance for further mechanistic investigations but also paves the way for potential development of new, safe, and cost-effective therapeutic strategies for TNBC.

## 1 Introduction

Breast cancer recently surpassed lung cancer as being the most diagnosed and leading cause of cancer deaths among women globally (1). The triple-negative breast cancer (TNBC) subtype accounts for approximately 15% of breast cancer cases but disproportionately causes breast cancer-related deaths (2). Chemotherapies remain the current mainstay treatment for TNBC, due to the lack of specific targets (3).

Although chemotherapies are effective for tumor debulking, they enrich a subpopulation of tumor cells known as cancer stem cells (CSCs). Enrichment of CD44^high^CD24^low^ and ALDH^high^ CSC populations in TNBC are attributed to treatment resistance and drug efflux, among other mechanisms (4). In addition, CSCs are capable of regenerating new tumors (4, 5).

Another feature of CSCs is their ability to evade anti-tumor immunity, as evidenced in literature (6–8). Despite remarkable progress in cancer immunotherapies, they remain costly and may contribute to autoimmunity and/or off-target toxicities (9, 10). Although beneficial for other cancer subtypes, the use of PD-L1 antibody atezolizumab, in combination with chemotherapy, was rescinded for advanced or metastatic TNBC due to the lack of benefit (11). Moreover, the role of current immunotherapeutic targets in the maintenance of CSCs has not been explored.

To this end, the 5’-ectonucleotidase CD73 has emerged as a novel target that promotes CSC survival and immune evasion of the tumor. Predominantly expressed on regulatory T cells, CD73 generates adenosine from its ligand, AMP, to inhibit immune effector functions. In the tumor microenvironment, the binding of adenosine may upregulate CSC-promoting pathways, although specific pathway crosstalk remains open to investigation (12–15). CD73-driven adenosinergic signaling is further exploited by TNBC to attenuate tumor-infiltrating T and NK cell activation (16, 17), and promote regulatory T cell functions (18). This is achieved mainly by the increase of intracellular cAMP levels in tumor-infiltrating lymphocytes, inducing a release in *IL-10* and *TGFβ* immune-suppressing cytokines. Interestingly, chemotherapies may directly upregulate CD73 activity and increase the availability of AMP ligand from the killing of bulk tumor cells (18–20).

As a demonstration of its therapeutic potential, CD73-deficient mice exhibit enhanced anti-tumor immunity, diminished tumor growth, and improved survival (21). The enzymatic activity of intracellular CD73 may also be important for tumor growth. To this point, small molecules have an advantage over anti-CD73 monoclonal antibodies, with the latter only targeting CD73 protein on the cell surface (22). Adenosine 5’-(α,β-methylene)diphosphate (APCP) is currently recognized as a competitive inhibitor for CD73. However, it only works at concentrations *in vitro* that are not translatable for clinical application (23). Therefore, identifying effective CD73 inhibitors is crucial for accelerating clinical translation.

In addition to intrinsic CD73 signaling in immunosuppression, key CSC-promoting pathways, namely Hippo and Wingless (Wnt), have also been attributed to CSC-mediated suppression of anti-tumor immunity (24–26). Inhibition of their major downstream effectors, Yes-associated protein (YAP) and β-catenin (β-cat), respectively, have been reported to suppress CSCs and simultaneously enhance anti-tumor immunity (27–29). Our previous findings identified the need to co-inhibit both YAP and Wnt for effective targeting of heterogenous CSC populations (30). Although promising, clinically applicable YAP and Wnt inhibitors are scarce, highlighting the need for uncovering novel pharmacological inhibitors.

Finding new compounds for clinical use remains challenging due to high cost, labor-intensive, and time-demanding processes (31). These obstacles may be mitigated with the use of high throughput *in silico* screening for the identification of drugs that may be repurposed for inhibiting CD73. In our study, we screened approximately 1000 natural compounds. Our results indicated certain flavonoids, presently used as over-the-counter supplements, are novel inhibitors of CD73, YAP, and Wnt. Notably, these compounds demonstrated inhibitor properties at clinically relevant doses. We developed a combinational therapy consisting of paclitaxel (a chemotherapeutic drug commonly used in the clinic), quercetin (found to be a CD73 inhibitor), and luteolin (found to be a Wnt/YAP dual inhibitor).

We found that this triple-drug combination inhibited bulk TNBC cells, antagonized paclitaxel-mediated enrichment of both CD44^high^CD24^low^ and ALDH1^high^ CSCs, and paclitaxel-upregulated/hyperactivated CD73. These findings were confirmed *ex vivo* using patient-derived xenograft (PDX) organotypic cultures that retain original tumor architecture and heterogeneity to represent clinical responses (32, 33). Lastly, we found this triple-drug combination improved frequencies of tumor-infiltrating lymphocytes *in vivo* using an immune-competent mouse model. Together, these findings offer a novel, effective, and clinically translatable avenue in developing treatments for TNBC.

## 2 Materials and methods

### 2.1 Cell culture and reagents

Cells were maintained at 37°C in a 5% CO_2_ incubator. Human MDA-MB-231 TNBC cells were purchased from ATCC (Manassas, VA, USA) and cultured in DMEM supplemented with 10% FBS and 1% penicillin/streptomycin. The E-cadherin^high^ MDA-MB-231 cell line variant was generated and maintained as described by Sulaiman et al. (30). Human SUM149-PT TNBC cells were purchased from Asterand (Detroit, MI, USA) and cultured in Ham’s F-12 supplemented with 5% FBS, 1% penicillin/streptomycin, 5 µg/mL insulin, 1 µg/mL hydrocortisone, and 10 mM HEPES. Mouse AT3 TNBC cells of C57BL/6 origin and transduced with retroviral vectors expressing chicken OVA cDNA (AT3^ova^) were purchased from the Peter MacCallum Cancer Institute (East Melbourne, AU). AT3^ova^ cells were maintained in DMEM supplemented with 10% FBS and 1% penicillin/streptomycin. Cells were routinely tested for mycoplasma using PCR. All relevant reagents and their sources are listed in **Supplementary Table 1**.

### 2.2 Ligand-receptor docking (*in silico*)

The docking software Molecular Operating Environment (MOE) purchased from Chemical Computing Group Inc (Montreal, QC, CA). The CD73 protein structure (accession code: 4H2G) was downloaded from Protein Data Bank and optimized for hydrogens and lone pairs within MOE. Natural compound structures were downloaded as potential ligands from PubChem in the SDF file format. The docking score, protein-ligand interactions, and the respective energies released from the interaction were generated and recorded using MOE Align/Superpose functions.

### 2.3 Reverse transcriptase and quantitative PCR (RT qPCR)

Cells were seeded into 6-well plates (3.0 x 10^5^ cells/well). Total RNA was extracted 72 hours after treatment using the Qiagen RNeasy kit (Toronto, ON, CA). cDNA was obtained from mRNA using the iScript cDNA Synthesis Kit purchased from Bio-Rad (Hercules, CA, USA), as per manufacturer instructions. Gene expression levels were determined using the Bio-Rad MyiQ real-time PCR system in a reaction mixture consisting of 50% SyBr Green, 37.5% RNAse-free water, 5% of forward/reverse primers, and 2.5% cDNA. Specific forward and reverse gene primers are listed in **Supplementary Table 2**. Results were normalized to *18S* or *GAPDH* housekeeping genes, and relative fold changes in gene expression was calculated using the ^2ΔΔ^CT method.

### 2.4 Flow cytometry

Cells were seeded into 6-well plates (3.0 x 10^5^ cells/well). Harvested cells were filtered through a 40 um strainer and suspended in PBS supplemented with 2% FBS and 2 mM EDTA 96 hours post-treatment. Non-specific binding was reduced with mouse anti-human IgG Fc from Thermo Fisher (Waltham, MA, USA) or rat anti-mouse CD16/CD32 from BD Biosciences (Franklin Lakes, NJ, USA). Cells were then incubated with fluorescently labeled antibodies (**Supplementary Table 3**). For human cells, apoptosis was determined 7-aminoactinomycin (7-AAD) purchased from eBioscience (San Diego, CA, USA). For mouse cells, apoptosis was determined prior to incubation with fluorescently labeled antibodies using the LIVE/DEAD fixable far red dead cell stain kit from Thermo Fisher (Waltham, MA, USA). ALDH activity was determined as per manufacturer instructions using the ALDEFLUOR kit from StemCell Technologies (Vancouver, BC, CA). Flow cytometry was performed on LSRFortessa (BD Biosciences) and data was analyzed using FloJo software (Ashland, OR, USA).

### 2.5 CD73 activity assay

Cells were seeded into 96-well plates (5.0 x 10^3^ cells/well). Cells were washed twice with pre-warmed phosphate-free buffer (distilled water supplemented with 2 mM MgCl_2_, 125 mM NaCl, 1 mM KCl, 10 mM glucose, and 10 mM HEPES up to pH 7.2) 48 hours post-treatment. Cells were then incubated at 37°C with 100 µM adenosine 5’-(α,β-methylene)diphosphate (APCP) or 250 µM AMP for 10 minutes. Resultant media phosphate concentrations were assessed as per manufacturer’s instructions using the Malachite Green Phosphate Detection Kit, purchased from R&D Systems (Minneapolis, MN, USA).

### 2.6 Cell viability assay

Cells were seeded into 24-well plates (1.5 x 10^3^ cells/well). A viability analysis was performed 120 hours post-treatment with a 3-hour incubation at 37°C using 10% 3-(4,5-Dimethylthiazol-2-Yl)-2,5-Diphenyltetrazolium Bromide (MTT, 1 mg/mL). The supernatant was aspirated, and formazan crystals were dissolved in DMSO. Resulting absorbance was measured at 570 nm.

### 2.7 Dual-luciferase reporter assay

Cells were seeded into 24-well plates (1.0 x 10^5^ cells/well). Cells were transfected with 1000 ng of either Wnt M50 Super 8x TOPFlash-luciferase (Addgene Plasmid #12456) or YAP 8xGTIIC-luciferase (Addgene Plasmid #34615), in conjunction with 1000 ng of Renilla pRL-SV40P (Addgene Plasmid #27163) using Lipofectamine 3000 purchased from Invitrogen (Carlsbad, CA, USA). Cells were treated 24 hours post-transfection and lysed for Firefly and Renilla luciferase activity 48 hours post-treatment using the Dual-Luciferase Reporter Assay System, purchased from Promega (Madison, WI, USA).

### 2.8 Tumorsphere formation assay

Cells were resuspended in 1:1 DMEM:F-12 supplemented with 2% B27, 1% sodium pyruvate, 1% penicillin/streptomycin, 20 ng/mL basic fibroblast growth factor, and 20 ng/mL epidermal growth factor and seeded into 96-well ultra-low attachment plates (1.5 x 10^3^ cells/well). Tumorspheres were counted (>100 µm diameter) and representative pictures were captured 10 days post-treatment using the Zeiss Axiovert 40 CFL microscope purchased from Carl Zeiss AG (Feldback, Switzerland). A cell viability assay was also performed as previously described.

### 2.9 Patient-derived xenograft organotypic cultures

The TNBC patient-derived xenograft samples HCI-001, HCI-002, HCI-015, and HCI-016 were obtained from and characterized by the University of Utah (33, 34). Extracted tumors were sliced with a scalpel to obtain 2 mm x 2 mm tumor fragments. These fragments were cultured in 48-well plates with 1:1 DMEM:F-12 supplemented with 10% FBS, 1 ug/mL insulin, 0.5 ng/mL hydrocortisone, 3 ng/mL epidermal growth factor, and 1% penicillin/streptomycin. Tumor fragments were treated, and viability was assessed daily with 10% resazurin sodium salt. Media fluorescence intensity was measured at 560 nm excitation and 530 nm emission following a 3-hour incubation at 37°C.

### 2.10 Mouse syngeneic tumor model (*in vivo*)

C57BL/6 wild-type (5-6 week old, female) mice were purchased from the Charles River Laboratory. Tumors were established in the mammary fat pads using 1 x 10^6^ AT3^ova^ cells resuspended in Matrigel purchased from BD Biosciences (Mississauga, ON, CA). Established AT3^ova^ tumors (3 mm x 3 mm) were treated intraperitoneally with paclitaxel (P, 10 mg/kg), luteolin (L, 20 mg/kg), quercetin (Q, 5 mg/kg), or adenosine 5’-(α,β-methylene)diphosphate sodium (APCP, 20 mg/kg) where indicated. Tumors were measured every 2 days using calipers. Mice were humanely euthanized once tumors reached a mean volume of 950 mm^3^. Tumors were minced with scissors and enzymatically digested with collagenase/hyaluronidase from StemCell Technologies (Vancouver, BC, CA) and DNAse I from Sigma Aldrich (St Louis, MO, USA).

### 2.11 Statistical analysis

Data are expressed as mean ± standard deviation (SD) or standard error (SE), where indicated, without data transformation and outlier exclusion. For relative comparison, the data were normalized to control group and then compared as indicated in each figure. Data distributions were tested by one-way ANOVA. When appropriate, statistical differences between groups were assessed by unpaired Student’s t-test or Mann-Whitney test (comparison of 2 groups). Statistical tests were completed using GraphPad Prism 9 software (GraphPad, San Diego, CA). Unless stated otherwise, experiments have a minimum of three biological repeats.

## 3 Results

### 3.1 Natural flavonoid quercetin targets CD73, a key mediator of immunosuppressive adenosine signaling commonly upregulated in TNBC

**Figure 1:**
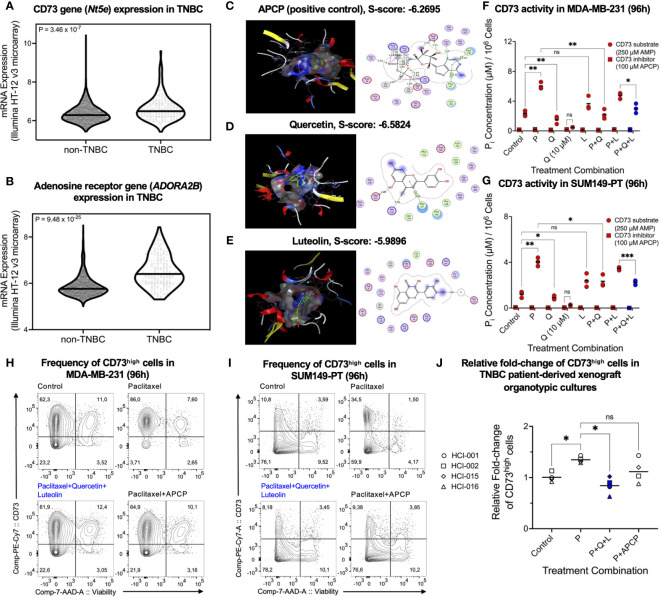
CD73^high^ cells and CD73 activity upregulated by paclitaxel are suppressed by quercetin. Relative expression levels of **(A)** *Nt5e* and **(B)** *ADORA2B* genes in 165 TNBC patients post-chemotherapy and 2344 non-TNBC patients post-treatment were compared using the METABRIC RNA-seq dataset on cBioPortal (www.cbioportal.org). Using the *in silico* drug design application Molecular Operating Environment (MOE), 2D/3D interactions and docking scores (S-score) between **(C)** adenosine 5’-(α,β-methylene)diphosphate sodium (APCP), **(D)** quercetin, and **(E)** luteolin and CD73 were visualized. Media phosphate levels, indicative of CD73 activity, were measured in **(F)** MDA-MB-231 and **(G)** SUM149-PT cells using the malachite green phosphate assay. Phosphate levels were assessed in the presence of CD73 inhibitor (APCP, 100 µM) or CD73 substrate (AMP, 250 µM) 48 hours post-treatment with paclitaxel (P, 2.5 nM), quercetin (Q, 0.5 or 10 µM), and luteolin (L, 5 µM) alone and in different combinations. Representative cell surface expression of CD73 was assessed 96 hours post-treatment with paclitaxel (2.5 nM), luteolin (5 µM), quercetin (0.5 µM), or APCP (2.5 µM) alone and in different combinations in **(H)** MDA-MB-231, **(I)** SUM149-PT, and **(J)** human TNBC patient-derived xenograft organotypic slice cultures. Data represents mean ± SD, n=3, ns, non-significant; *, P<0.05; **, P<0.01; ***, P<0.005.

Targeting enriched CSCs in TNBC post-chemotherapy remains challenging, owing to limited knowledge of immunotherapeutic targets. To identify novel targets, we adopted the METABRIC RNA-seq dataset to compare 165 TNBC samples post-chemotherapy with 2344 non-TNBC breast cancer samples post-treatment (chemotherapy/hormone therapy/radiotherapy). Components of the adenosinergic signaling pathway were identified as potential clinical targets, including CD73 (**Figure 1A**) and adenosine receptor ADORA2B (**Figure 1B**). Given the limitation of current CD73 inhibitors for clinical translation, we used quantitative structure-activity relationships (QSAR) from high throughput *in silico* molecular docking between CD73 and natural compounds for virtual screening. Using the CD73 inhibitor APCP as a positive control (**Figure 1C**), we found that the flavonoids quercetin (**Figure 1D**) and luteolin (**Figure 1E**) exhibited relatively high docking scores, indicating a notable specificity for CD73 (PDB# 4H2G), with quercetin yielding extensive molecular interactions (**Supplementary Table 4**). Of note, quercetin and luteolin have been safely used as over-the-counter medicine/supplements.

To validate our *in silico* findings *in vitro*, we tested clinically relevant doses of quercetin (35) and luteolin (36) with the chemotherapeutic, paclitaxel, on CD73 activity. By measuring media phosphate concentrations normalized to cell count in the presence of AMP substrate, we found that 10 µM quercetin potently inhibited paclitaxel-upregulated CD73 activity, comparable to paclitaxel combined with 100 µM APCP in human TNBC MDA-MB-231 (**Figure 1F**) and SUM149-PT (**Figure 1G**) cell lines. Strikingly, quercetin, but not luteolin, antagonized the paclitaxel-mediated upregulation of CD73 activity (**Figures 1F, G**), highlighting the efficacy of quercetin as a clinically translatable CD73 inhibitor.

Next, we employed flow cytometric analysis to confirm that the observed reduction in CD73 activity by quercetin reduced paclitaxel-enriched CD73 protein expression in TNBC cells. The CD73 inhibitor APCP in combination with paclitaxel was used as a positive control. We found that quercetin did not lower CD73 expression but reduced the frequency of living CD73^high^ cells alone and antagonized the paclitaxel-mediated enrichment of living CD73^high^ cells in combination (**Figures 1H, I**; **Supplementary Figures S1A, B**). Similar results were obtained from the chemo-resistant mouse AT3^ova^ TNBC cells (**Supplementary Figure S1C**) and TNBC PDX organotypic cultures (**Figure 1J**), implying that clinically achievable doses of quercetin may effectively reduce CD73^high^ tumor cells post-chemotherapy.

### 3.2 Combination of quercetin, luteolin, and paclitaxel synergistically suppresses viability of human TNBC cell lines and PDX organotypic cultures

**Figure 2:**
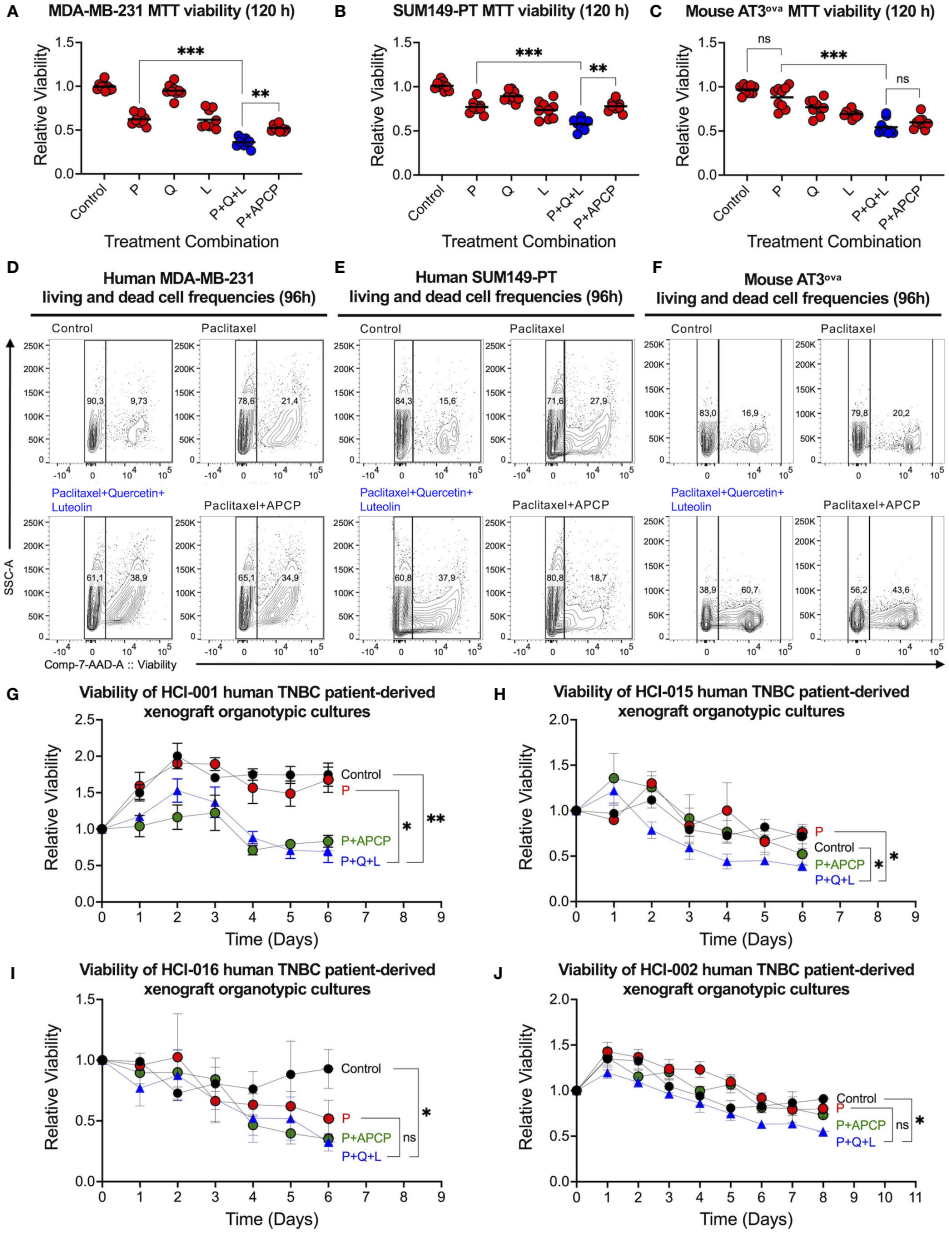
Triple-drug combination effectively suppresses bulk human TNBC cells. Viability of **(A)** MDA-MB-231 and **(B)** SUM149-PT human TNBC, and **(C)** AT3^ova^ mouse TNBC cell lines were assessed in an MTT assay 120 hours post-treatment with paclitaxel (P, 2.5 nM), quercetin (Q, 0.5 µM), luteolin (L, 5 µM), and adenosine 5’-(α,β-methylene)diphosphate (APCP, 5 µM) alone and in different combinations. Representative flow cytometric analysis of **(D)** MDA-MB-231, **(E)** SUM149-PT, and **(F)** AT3^ova^ cells 96 hours post-treatment with DMSO control, paclitaxel, triple-drug combination, and paclitaxel with APCP using 7-AAD to assess apoptotic/dead cells. Viabilities of chemo-resistant **(G)** HCI-001, **(H)** HCI-015 and chemo-sensitive **(I)** HCI-016 and **(J)** HCI-002 TNBC patient-derived xenograft organotypic slice cultures were assessed daily using the alamar blue assay. Data represents mean ± SD, n=3, ns, non-significant; *, P<0.05; **, P<0.01; ***, P<0.001.

Given that luteolin did not inhibit CD73 activity, we then tested its inhibitory efficacy on TNBC cell viability to validate the three-drug combination. Paclitaxel used in the clinical treatment of TNBC, and paclitaxel combined with CD73 inhibitor APCP were taken as positive controls. The combination of quercetin, luteolin, and paclitaxel was most effective at killing human MDA-MB-231 (**Figure 2A**) and SUM149-PT (**Figure 2B**), and chemo-resistant mouse AT3^ova^ (**Figure 2C**) TNBC cell lines in MTT viability assays. The triple-drug combination also reduced cell viability more significantly than any other dual-combinations (**Supplementary Figures S2A–C**). In contrast, the inclusion of quercetin and luteolin in the triple-drug combination did not lead to a reduction in the viability of MCF10a cells (a non-tumorigenic mammary cell line) beyond that observed with paclitaxel alone (**Supplementary Figure S2D**). Interestingly, the chemo-resistant mouse AT3^ova^ cells were sensitized to treatment, mainly by luteolin. Using the Chou-Talalay method, synergism was also observed in the triple-drug combination using the clinically relevant doses of each drug (**Supplementary Figures S2E, F**).

Since MTT cell viability assays are metabolic activity-dependent, we performed flow cytometry and confirmed that the triple-drug combination was the most effective in increasing frequencies of late apoptotic and dead TNBC cells (**Figures 2D–F**). Furthermore, we found that clinically relevant doses of the triple-drug combination significantly reduced *ex vivo* viability of four distinct TNBC PDX organotypic cultures compared to control or paclitaxel alone (**Figures 2G–J**). Of note, chemo-resistant PDX samples were sensitized to triple-drug treatment and exhibited significant reductions in viability (**Figures 2G, H**). Together, these data suggest that luteolin played an important role in the triple-drug combination by promoting TNBC cell killing *via* other mechanisms associated with treatment resistance post-chemotherapy.

### 3.3 Combination of quercetin, luteolin, and paclitaxel represses YAP and Wnt cancer stem cell-promoting pathways

**Figure 3:**
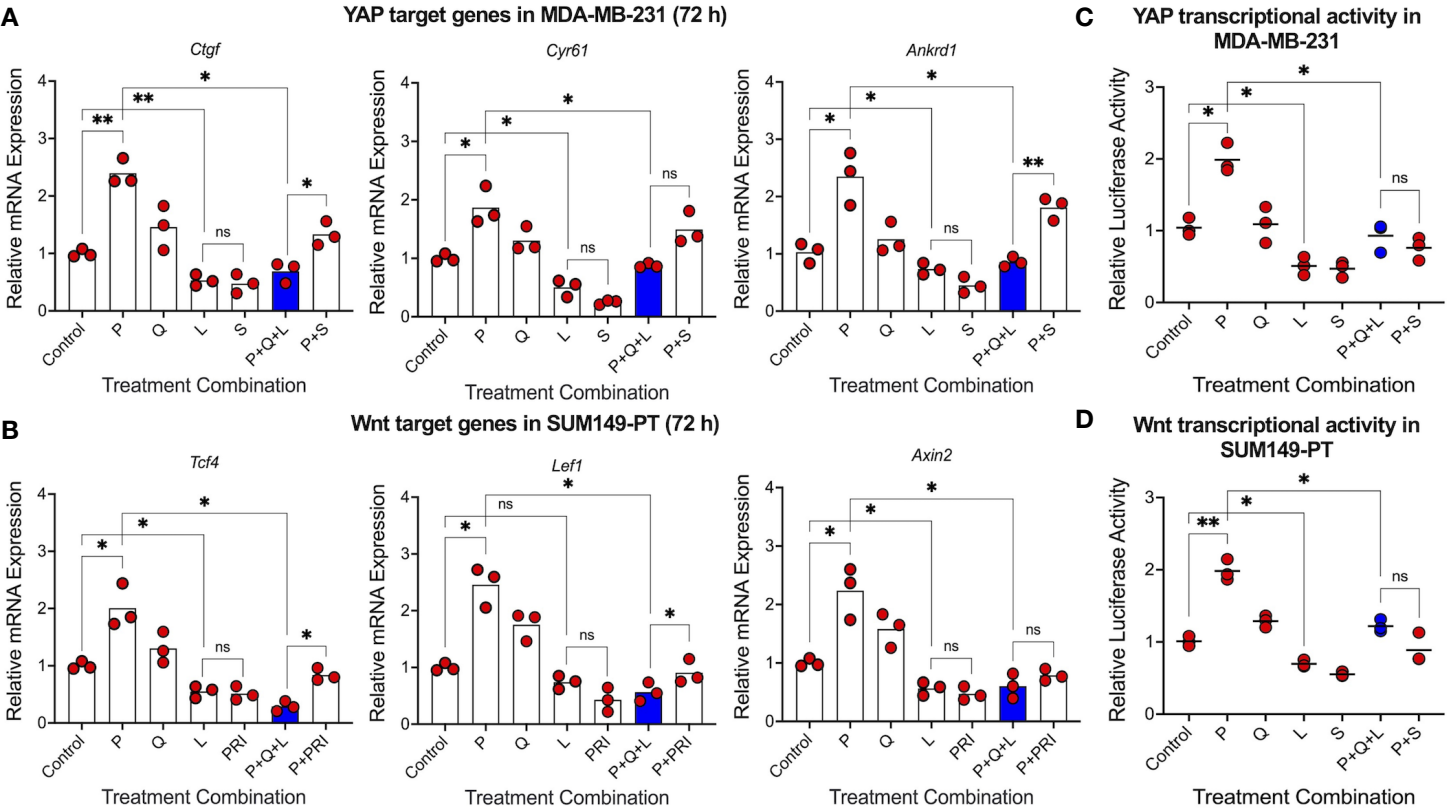
Triple-drug combination effectively suppresses cancer stem cell-promoting YAP and Wnt signaling pathways. RT-qPCR analysis was carried out on **(A)** YAP target genes in MDA-MB-231 cells and **(B)** Wnt target genes in SUM149-PT cells 72 hours post-treatment with paclitaxel (5 nM), quercetin (1 µM), and luteolin (10 µM) alone and in different combinations. Simvastatin (500 nM) and PRI-724 (5 µM) were used as positive controls for YAP and Wnt inhibition, respectively. Transcriptional activity of **(C)** YAP and **(D)** Wnt were also assessed using the dual-luciferase reporter assay 48 hours post-treatment with paclitaxel (P, 5 nM), quercetin (Q, 1 µM), luteolin (L, 10 µM), simvastatin (S, 250 nM), and PRI-724 (PRI, 2.5 µM) alone and in different combinations. Data represents mean ± SD, n=3, ns, non-significant; *, P<0.05; **, P<0.01.

Since CSCs are crucial for tumor relapse, we then asked whether triple-drug combination could suppress two key CSC-promoting pathways, YAP and Wnt, that have been closely linked to the maintenance and enrichment of mesenchymal-like and epithelial-like CSCs, respectively (28, 37–39). Gene expression analysis showed that luteolin significantly reduced expression of YAP target genes *Ctgf*, *Cyr61* and *Ankrd1* in MDA-MB-231 human TNBC cells, comparable to simvastatin, a known YAP inhibitor (**Figure 3A**). Similarly, luteolin significantly reduced expression of Wnt target genes *Tcf4*, *Lef1*, and *Axin2* in SUM149-PT human TNBC cells, comparable to PRI-724, a known Wnt inhibitor (**Figure 3B**). In both instances, luteolin but not quercetin antagonized the paclitaxel-mediated upregulation of those target genes (**Supplementary Figures S3A, B**). To consolidate these findings, we performed dual-luciferase reporter assays to assess transcriptional activity of YAP and Wnt. We found significant reductions in the transcriptional activity of both CSC-promoting pathways by the triple-drug combination in human MDA-MB-231 (**Figure 3C**) and SUM149-PT (**Figure 3D**) TNBC cells, respectively. These results imply that triple-drug combination may not only suppress CD73 but also mesenchymal-like and epithelial-like CSCs.

### 3.4 Combination of quercetin, luteolin, and paclitaxel antagonizes paclitaxel-mediated enrichment of mesenchymal-like and epithelial-like CSCs

**Figure 4:**
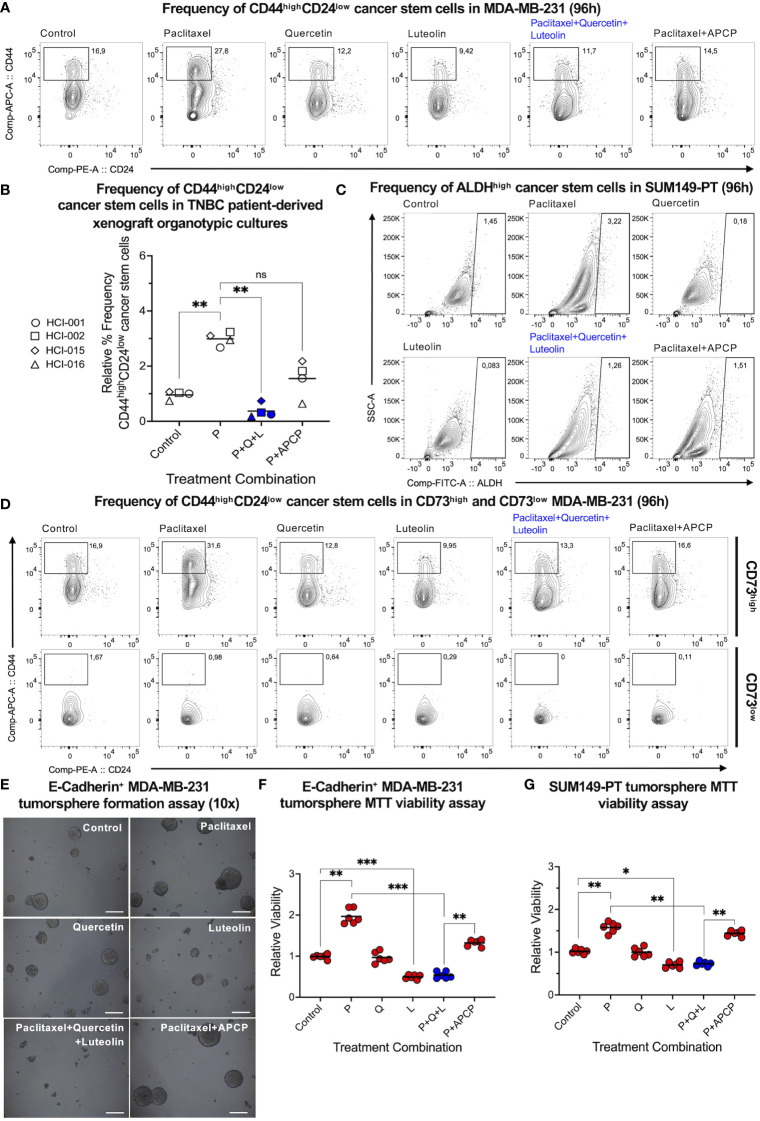
Co-inhibition of CD73, YAP, and Wnt effectively suppresses paclitaxel-upregulated TNBC cancer stem cells. Flow cytometric analysis was performed to assess CD44^high^CD24^low^ cancer stem cells post-treatment on **(A)** MDA-MB-231 and **(B)** TNBC patient-derived xenograft tumor chunks. **(C)** ALDH^high^ cancer stem cell markers were also assessed on SUM149-PT cells post-treatment. All cells were treated with paclitaxel (P, 2.5 nM), quercetin (Q, 0.5 µM), luteolin (L, 5 µM), and adenosine 5’-(α,β-methylene)diphosphate (APCP, 5 µM) alone and in different combinations. **(D)** Frequencies of CD44^high^CD24^low^ cancer stem cells were reassessed following CD73^high^ versus CD73^low^ gating in MDA-MB-231 cells. MDA-MB-231 cells were transduced with a lentiviral vector encoding the E-cadherin gene insert. Epithelial-like E-cadherin^+^ MDA-MB-231 cells were grown in non-adherent and serum-free conditions for tumorsphere formation. Tumorspheres were treated with paclitaxel (P, 2.5 nM), quercetin (Q, 0.5 µM), luteolin (L, 5 µM), and APCP (10 µM) alone and in different combinations. **(E)** Representative pictures (scale bar = 100 µm) were captured on Day 10 of tumorsphere formation; **(F)** an MTT assay was performed to corroborate tumorsphere viability. **(G)** The latter was repeated on epithelial-like SUM149-PT cells. Data represents mean ± SD, n=3, ns, non-significant; *, P<0.01; **, P<0.005; ***, P<0.0005.

Next, we hypothesized that quercetin and luteolin would antagonize the paclitaxel-mediated enrichment of CD44^high^CD24^low^ mesenchymal-like and ALDH1^high^ epithelial-like CSC populations (4). Through flow cytometric analysis, we found that the triple-drug combination circumvented the paclitaxel-mediated enrichment of CD44^high^CD24^low^ CSCs in mesenchymal-like MDA-MB-231 cells (**Figure 4A**) and PDX organotypic cultures (**Figure 4B**). The same trend was observed with ALDH^high^ CSCs in epithelial-like SUM149-PT cells (**Figure 4C**) and mouse AT3^ova^ TNBC cells (**Supplementary Figure S3C**). Interestingly, quercetin in combination with paclitaxel was unable to reduce paclitaxel-mediated CSC enrichment as effectively as APCP in combination with paclitaxel (**Supplementary Figures S3D, E**).

Since CD73 has been related to CSC survival, we gated on CD73^high^ and CD73^low^ MDA-MB-231 cells to further understand the association between CD73 and CSCs. Interestingly, significantly higher frequencies of CD44^high^CD24^low^ CSCs were observed in CD73^high^, but not in CD73^low^ populations, irrespective of the different treatments (**Figure 4D**). This suggested that CD73 itself may be a promoter of mesenchymal-like CSCs. Given very low expression of CD73 in epithelial-like TNBC cells, we tested the effect of quercetin, luteolin, and CD73 inhibitor APCP on epithelial-like tumorsphere formation (an *in vitro* functional assay to assess CSCs). Quercetin and luteolin were observed to reduce the paclitaxel-enhanced tumorsphere formation in human MDA-MB-231 over-expressing E-cadherin (**Figures 4E, F**) and SUM149-PT (**Figure 4G**) epithelial-like TNBC cells, implying inhibition of CSC functions *in vitro*. In contrast, CD73 inhibitor APCP did not antagonize paclitaxel-mediated enrichment in epithelial-like tumorspheres, thereby emphasizing the potential benefit of quercetin, luteolin, and paclitaxel in the inhibition of CSCs *in vitro*.

### 3.5 Combination of quercetin, luteolin, and paclitaxel reduces tumor mass and CSC frequencies while boosting anti-tumor immunity in C57BL/6 mice

**Figure 5:**
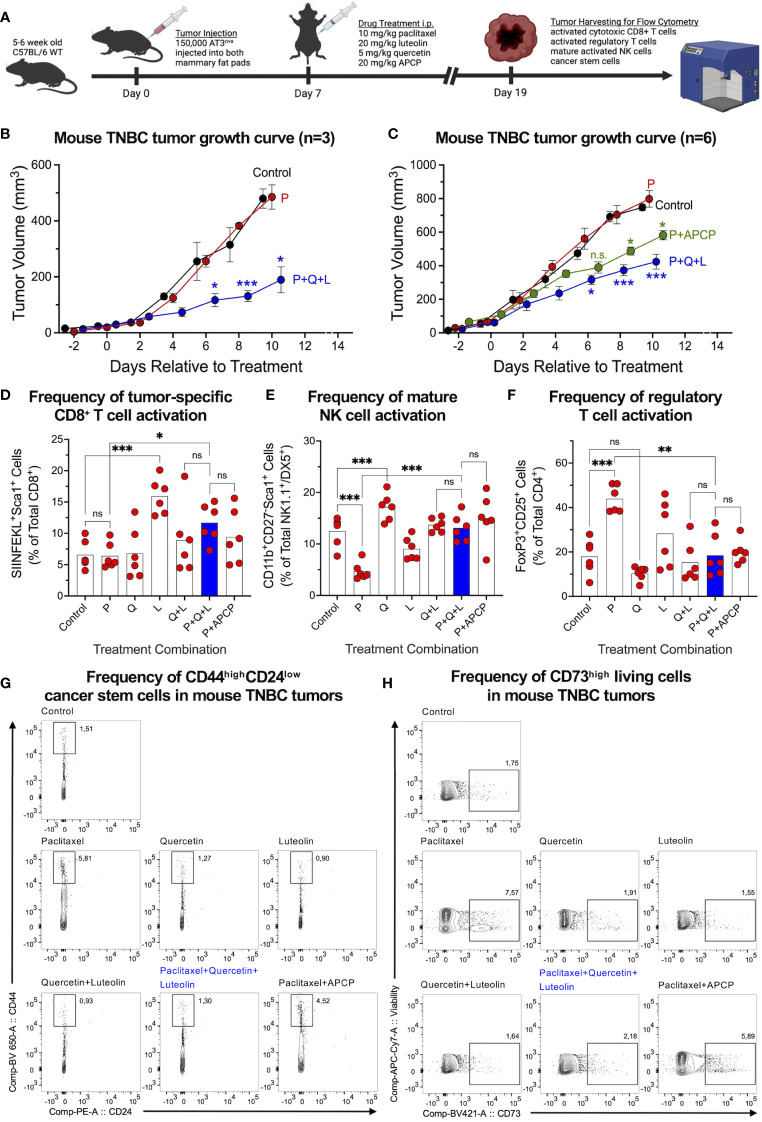
Triple-drug treatment effectively suppresses TNBC tumors and simultaneously improves frequencies of tumor-infiltrating lymphocytes. **(A)** AT3^ova^ mouse TNBC tumors were established in the mammary fat pads of C57BL/6 mice which were randomized into treatment cohorts. **(B)** Tumor dimensions were measured every second day to assess tumor growth in response to treatment. **(C)** Experiment was repeated to include more treatment groups and analysis of tumor infiltrating lymphocytes. Flow cytometric analysis revealed an increase in the frequencies of **(D)** tumor-specific activated CD8^+^ T cells and **(E)** mature activated NK cells, and a decrease in **(F)** activated regulatory T cells in response to the triple-drug combination relative to chemotherapy alone. **(G)** Cancer stem cell analysis on mouse tumor samples using CD44^high^CD24^low^ markers outlined significant improvements by the triple-drug combination relative to paclitaxel alone. **(H)** Similar trends were observed for CD73 expression on tumor cells in response to treatment. Data represents mean ± SEM **(B, C)**; mean ± SD **(D–F)**, n=6, ns, non-significant; *, P<0.05; **, P<0.005; ***, P<0.001 by two-way ANOVA.

To investigate whether the triple-drug combination would maintain superior treatment potential within a heterogenous tumor microenvironment, we injected chicken ovalbumin (ova)-expressing (AT3^ova^) mouse TNBC cells into the mammary fat pads of syngeneic immune-competent C57BL/6 mice, followed by treatments (**Figure 5A**). We found that the triple-drug treatment significantly inhibited the growth of chemo-resistant AT3^ova^ tumors (**Figure 5B**). We repeated the experiment to further investigate the effect of each drug alone on tumor growth, with APCP in combination with paclitaxel as a positive control. The triple-drug combination was much more effective than APCP combined with paclitaxel at reducing tumor growth (**Figure 5C**). Moreover, quercetin and luteolin in combination were more effective at reducing tumor growth than each drug alone; notably, none of the treatments impacted mouse body weight **(Supplementary Figures S4A, B**).

Next, we investigated the frequencies of tumor-infiltrating lymphocytes, given the observations that CD73 inhibits CD8^+^ T and NK cells while upregulating CD4^+^ regulatory T cell activities (16–18). The OVA-expressing tumors allowed for the assessment of tumor-specific CD8^+^ T cell responses using the ovalbumin peptide SIINFEKL loaded tetramers. We found that the triple-drug combination markedly increased the frequency of tumor-specific activated CD8^+^ T cells (**Figure 5D**) and mature activated NK cells that were suppressed following paclitaxel treatment (**Figure 5E**). Of note, luteolin alone was the significant contributor to a higher frequency of tumor-specific activated CD8^+^ T cells, whereas quercetin alone was the significant contributor to a higher frequency of activated mature NK cells. We also found that the triple-drug combination reduced the paclitaxel-mediated increase in the frequencies of immune-suppressing CD4^+^ regulatory T cells (**Figure 5F**). CD73 expression on activated regulatory T cells was also reduced significantly by quercetin and its upregulation by paclitaxel was antagonized by the triple-drug combination (**Supplementary Figure S4C**). Consistent with our *in vitro* findings, paclitaxel alone enriched the frequency of CD44^high^CD24^low^ CSCs, which was averted mainly by luteolin alone and in combination (**Figure 5G**). A similar trend in response to the treatment was observed among CD73^high^ tumor cells (**Figure 5H**).

## 4 Discussion

CSCs and their ability to evade anti-tumor immunity contribute to the aggressive nature of TNBC (4), while targeting CSCs using small molecules have yet to be developed. Although there are ongoing early phase clinical trials involving CD73 monoclonal antibodies (40), they face challenges such as inadequate tumor penetration and the inability to target intracellular CD73. Furthermore, the current pre-clinical small molecule inhibitor, APCP, demands high doses that may lead to intolerable toxicity (23). To address this concern, we investigated flavonoids that have been consumed as over-the-counter supplements and exhibit several advantages for the development of cancer therapies, such as relatively low toxicities, cost-effectiveness, and high commercial availability (41, 42). Drug repurposing through *in silico* screening allow for the bypass of high costs, labor-intensive, and time-consuming processes associated with conventional drug discovery (31).

In addition to exhibiting *in silico* docking specificity for CD73, quercetin and luteolin used in this study were stable and demonstrated plasma concentrations achievable in a clinical setting (43–46). Both quercetin and luteolin are commonly used as over-the-counter supplements and are recorded to have excellent safety profiles with no reported serious adverse effects (47–49). This was consistent with our findings in which the triple-drug treatment did not reduce viability of MCF10a non-tumorigenic breast cells relative to paclitaxel alone. Although we have high confidence in the safety of combining quercetin and luteolin with paclitaxel, further *in vivo* studies may be required to determine whether the combination would influence the bioavailability of other drugs.

The role of chemotherapeutics such as paclitaxel in enriching CD73^high^ cells in both mesenchymal-like and epithelial-like cancer cell populations and upregulating CSC-promoting pathways such as YAP or Wnt have limited its treatment efficacy (4). Contrary to our *in silico* findings, we showed that quercetin but not luteolin antagonized the paclitaxel-mediated enrichment of CD73, which may be associated with its potential targeting of upstream transcriptomic regulators of CD73, such as c-Jun/AP-1 (50–53). In contrast to its prominent role in CD73 inhibition, quercetin had insignificant inhibitory effects on both YAP and Wnt CSC-promoting pathway. Given the potential activation of Wnt signaling downstream of CD73-mediated adenosinergic signaling (14, 15), the suppression of CD73^high^ cells by quercetin alone may not strongly translate to an inhibition of downstream signaling in CSCs. While our *in silico* and *in vitro* findings support and align with the specificity of quercetin for CD73 (54, 55), there remains a limited understanding of its exact molecular targets, thus warranting further investigation into the selectivity of quercetin for CD73.

Of note, we found that luteolin but not quercetin effectively inhibited both YAP and Wnt CSC-promoting signaling pathways, alone and in the triple-drug combination, partially explaining the observed reductions in CSC frequencies at a clinically achievable concentration. While the mechanistic interactions between CD73 and CSC-promoting pathways remains unclear, we found that CD73 expression is highly correlated with mesenchymal-like CSCs, but not non-CSCs. CD73 has shown to exhibit intrinsic roles in promoting epithelial-to-mesenchymal transition (14), partially explaining our results that paclitaxel and APCP in combination increased tumorsphere formation. It is possible that CD73 inhibition without co-inhibiting CSC-promoting pathways may facilitate proliferation of epithelial-like CSCs.

Our results suggest that the three-drug combination, using clinically relevant doses of quercetin and luteolin, is an effective approach to simultaneously inhibit paclitaxel-enriched CD73 and CSCs in TNBC. This approach may also be effective for overcoming treatment resistance, as several studies have shown that chemotherapy-enriched CSCs often fail to respond to subsequent treatments (56, 57). We used the CD73 inhibitor APCP as a control for quercetin, and the YAP and Wnt inhibitors, simvastatin (a repurposed clinical drug) and PRI-724 (in clinical trials) as controls for luteolin. Of note, we found quercetin exhibited greater efficacy than APCP at suppressing CD73 activity and expression, while luteolin exhibited equivalent efficacy as simvastatin and PRI-724 in suppressing YAP and Wnt CSC-promoting pathways. Luteolin has shown to inhibit other pathways associated with CSC promotion (58–60). However, it remains unclear whether the three-drug combination also suppresses interactions between CD73 and other CSC-promoting pathways.

To further emphasize on clinical translatability of the triple-drug combination, we performed experiments using *ex vivo* PDX organotypic cultures using clinically achievable concentrations and obtained similar results. Resemblance between the viability and CSC frequencies in organotypic cultures and actual clinical responses support potential benefit of the triple-drug combination in the treatment of TNBC. Since CSCs remain known contributors to treatment resistance in TNBC, the ability of the triple-drug combination to suppress CSCs in PDX organotypic cultures implied its clinical potential.

Given the important role of CD73 in suppressing anti-tumor immunity (16–18), we explored the role of the natural flavonoids and triple-drug treatment using a TNBC immune-competent C57BL/6 mouse model. The injection of mouse AT3^ova^ cells into the mouse mammary fat pats, along with intraperitoneal drug administration, provided a suitable model of TNBC (61), as it allows for the hepatic metabolism of drugs prior to systemic circulation. Quercetin but not luteolin increased mature NK activation, which may be partially explained by the direct inhibition of NK functions by CD73-generated adenosine (16). On the other hand, luteolin but not quercetin increased tumor-specific CD8^+^ T cell activation, which may be partially due to inhibition of YAP/Wnt-induced suppression of cytotoxic T cell responses rather than tumor-mediated adenosinergic signaling (62, 63). Interestingly, the frequency of CD73^high^ cells following paclitaxel treatment increased considerably more on tumor cells than regulatory T cells, suggesting a higher vulnerability of tumor cells in response to chemotherapy. Similar to our *in vitro* findings, the triple-drug treatment was more effective than paclitaxel in combination with APCP at reducing CSC frequencies, implying that CD73 inhibition alone might be insufficient for tumor control.

Moving forward, investigating the interplay between CD73 and CSC-promoting pathways may strengthen the understanding of TNBC immune evasion. Specifically, the adenosine receptors in TNBC may exhibit specific methylation patterns (64), potentially amplifying their activity and promoting CSC survival. While our work provided insight into the influence of CD73 and CSCs on tumor-infiltrating lymphocytes, future studies might involve investigating metastatic tumors post-treatment as a measure of disease recurrence (61, 65), or employing humanized mouse models to evaluate translatability of our findings regarding tumor-infiltrating human lymphocytes (66). Moreover, recent preclinical studies have indicated that bivalent CD73/PD-1 antibodies significantly improve the activation of tumor-infiltrating lymphocytes (67, 68), implying that our triple-drug treatment might enhance treatment response if combined with FDA-approved anti-PD-1 therapies such as pembrolizumab. Additionally, the exploration of nano-molecularly imprinted polymers (nano-MIPs) or other nanotechnologies to enhance tumor-specific drug delivery could be investigated to improve activation of tumor-infiltrating lymphocytes (69).

Taken together, our results show that each natural flavonoid in the triple-drug combination plays a unique yet important role in targeting TNBC. By screening the efficacy and function of each drug alone and in various combinations using clinically relevant doses, we identified luteolin and quercetin as potent antagonists of paclitaxel-enriched CD73 and CSCs, and their combination with paclitaxel effectively reducing TNBC tumor growth and promoting anti-tumor immunity *in vivo*. In conclusion, this study integrated *in silico*, *in vitro*, and *in vivo* findings to propose a novel, cost-effective, clinically translatable approach for targeting CSCs and improving anti-tumor immunity, thereby increasing the potential of significantly improving clinical outcomes for patients with TNBC.

## Data availability statement

The original contributions presented in the study are included in the article/**Supplementary Material**, further inquiries can be directed to the corresponding authors.

## Ethics statement

Ethical approval was not required for the studies on humans in accordance with the local legislation and institutional requirements because only commercially available established cell lines were used. The animal study was approved by University of Ottawa Animal Care Committee. The study was conducted in accordance with the local legislation and institutional requirements.

## Author contributions

KM: Conceptualization, Data curation, Formal analysis, Investigation, Methodology, Project administration, Software, Validation, Visualization, Writing – original draft, Writing – review & editing. SE-S: Investigation, Methodology, Writing – original draft, Writing – review & editing. MM: Investigation, Methodology, Writing – original draft, Writing – review & editing. MZA: Investigation, Methodology, Writing – original draft, Writing – review & editing. MK: Investigation, Methodology, Writing – original draft, Writing – review & editing. ASa: Investigation, Methodology, Writing – original draft, Writing – review & editing. RK: Investigation, Methodology, Writing – original draft, Writing – review & editing. SA: Investigation, Methodology, Writing – original draft, Writing – review & editing. ASh: Investigation, Methodology, Writing – original draft, Writing – review & editing. CC: Investigation, Methodology, Writing – original draft, Writing – review & editing. ASu: Investigation, Methodology, Writing – original draft, Writing – review & editing. SM: Investigation, Methodology, Writing – original draft, Writing – review & editing. JL: Writing – original draft, Writing – review & editing. ZL: Writing – original draft, Writing – review & editing. S-HL: Writing – original draft, Writing – review & editing. XL: Writing – original draft, Writing – review & editing. GS: Writing – original draft, Writing – review & editing. VD’C: Writing – original draft, Writing – review & editing. MA: Conceptualization, Data curation, Formal analysis, Funding acquisition, Investigation, Methodology, Project administration, Resources, Software, Supervision, Validation, Visualization, Writing – original draft, Writing – review & editing. LW: Conceptualization, Data curation, Formal analysis, Funding acquisition, Investigation, Methodology, Project administration, Resources, Software, Supervision, Validation, Visualization, Writing – original draft, Writing – review & editing.
