# Supplementary material for: Targeting CD73 with flavonoids inhibits cancer stem cells and increases lymphocyte infiltration in a triple-negative breast cancer mouse model
Source: Front Immunol. 2024 Mar 27;15:1366197. doi: 10.3389/fimmu.2024.1366197 (PMC11004431; doi:10.3389/fimmu.2024.1366197)
Supplement: Supplementary file 1 [file DataSheet_1.docx]

***Supplementary Material***

**Targeting CD73 with flavonoids inhibits cancer stem cells and increases lymphocyte infiltration in a triple-negative breast cancer mouse model.**

**Karan Mediratta, Sara El-Sahli, Marie Marotel, Muhammad Z. Awan, Melanie Kirkby, Ammar Salkini, Reem Kurdieh, Salman Abdisalam, Amit Shrestha, Chiara Di Censo, Andrew Sulaiman, Sarah McGarry, Jessie R. Lavoie, Zhen Liu, Seung-Hwan Lee, Xuguang Li, Giuseppe Sciume, Yanqi Dang, Wenjun Zhou, Guang Ji, Vanessa M. D'Costa, Michele Ardolino*, Lisheng Wang***

*** Correspondence:**

Michele Ardolino - [m.ardolino@uottawa.ca](mailto:m.ardolino@uottawa.ca); Lisheng Wang - [Lisheng.Wang@uottawa.ca](mailto:Lisheng.Wang@uottawa.ca)

**Supplementary Table 1. List of cell culture reagents and resources.**

| **Reagent** | **Source** | **Identifier** |
| --- | --- | --- |
| DMEM | Wisent | Cat No. 319-015-CL |
| Ham’s F-12 | Wisent | Cat No. 318-010-CL |
| Adenosine 5'-(α,β-methylene)diphosphate (APCP) | Sigma-Aldrich | Item No. M3763 |
| Adenosine monophosphate (AMP) | Sigma-Aldrich | Item No. A1752 |
| B27 | Gibco | Cat No. 17504044 |
| Basic fibroblast growth factor | R&D Systems | Cat No. 234-FSE |
| Epidermal growth factor | R&D Systems | Cat No. 236-EG |
| Fetal bovine serum (FBS) | Wisent | Cat No. 080450 |
| Hydrocortisone | Sigma-Aldrich | Item No. H6909 |
| Insulin | Sigma-Aldrich | Item No. 91077C |
| Luteolin (L) | Cayman Chemical | Item No. 10004161 |
| Paclitaxel (P) | Cayman Chemical | Item No. 10461 |
| Penicillin/Streptomycin | HyClone | Cat No. SV30010 |
| Quercetin (Q) | Cayman Chemical | Item No. 10005169 |
| Resazurin sodium salt | Cayman Chemical | Item No. 14322 |
| Sodium pyruvate | Gibco | Cat No. 11360070 |
| Thiazolyl Blue Tetrazolium Bromide (MTT) | Sigma-Aldrich | Item No. M2128 |

**Supplementary Table 2. List of forward and reverse primers used for the assessment of genes expression in human or mouse TNBC cells by RT-qPCR.**

| **Gene** | **Primer Sequence** | |
| --- | --- | --- |
|  | **Forward** | **Reverse** |
| *Gapdh* | AATGGGCAGCCGTTAGGAAA | GCGCCCAATACGACCAAATC |
| *18s* | AACCCGTTGAACCCCATT | CCATCCAATCGGTAGTAGCG |
| *Nt5e* (human) | GCCTGGGAGCTTACGATTTTG | ATAGTGCCCTGGTACTGGTC |
| *Nt5e* (mouse) | GAAACCTGATCTGTGATGCCA | TGCCACCTCCGTTTACAATG |
| *Ctgf* | AGGAGTGGGTGTGTGACGA | CCAGGCAGTTGGCTCTAATC |
| *Cyr61* | AGCCTCGCATCCTATACAACC | TTCTTTCACAAGGCGGCACTC |
| *Ankrd1* | CACTTCTAGCCCACCCTGTGA | CCACAGGTTCCGTAATGATTT |
| *Tcf4* | GAGGTGGCATGCACTGTC | CGCTGTGTTCATTGGTCTCT |
| *Lef1* | TTCTCCACCCATCCCGAGAAC | CTGAGGCTTCACGTGCATT |
| *Axin2* | GTCACCAAACCCATGCCTGTCTCT | TAAGCACCGTCTTGATCGCCCAAT |

**Supplementary Table 3. List of fluorescent antibodies used for the assessment of cell surface marker expression in human or mouse TNBC cells or tumors by flow cytometry.**

| **Antibody** | **Source** | **Identifier** |
| --- | --- | --- |
| Alexa Fluor 647 Rat anti-Mouse Foxp3 | BD Biosciences | Cat No. 560402 |
| Alexa Fluor 700 Hamster anti-Mouse CD69 | BD Biosciences | Cat No. 561238 |
| Alexa Fluor 700 Rat anti-Mouse CD4 | BD Biosciences | Cat No. 557956 |
| APC Mouse anti-Human CD44 | BD Biosciences | Cat No. 559942 |
| BV421 Rat anti-Mouse CD8a | BD Biosciences | Cat No. 563898 |
| BV510 Rat anti-Mouse Ly-6A/E (Sca1) | BD Biosciences | Cat No. 744323 |
| BV605 Rat anti-Mouse CD11b | BD Biosciences | Cat No. 563015 |
| BV650 Rat anti-Mouse CD44 | BD Biosciences | Cat No. 740455 |
| BV711 Hamster anti-Mouse CD3e | BD Biosciences | Cat No. 740665 |
| FITC Mouse anti-Mouse CD45.2 | BD Biosciences | Cat No. 561874 |
| FITC Rat anti-Mouse CD62L | BD Biosciences | Cat No. 561917 |
| PE Mouse anti-Human CD24 | BD Biosciences | Cat No. 555428 |
| PE Mouse anti-Mouse NK-1.1 | BD Biosciences | Cat No. 557391 |
| PE OVA257-264 (SIINFEKL) peptide bound to H-2Kb | Thermo Fisher | Cat No. 12-5743-81 |
| PE Rat anti-Mouse CD24 | BD Biosciences | Cat No. 561079 |
| PE Rat anti-Mouse CD25 | BD Biosciences | Cat No. 561065 |
| PE-CF594 Mouse anti-Mouse CD45.2 | BD Biosciences | Cat No. 565390 |
| PE-CF594 Rat anti-Mouse CD49b (DX5) | BD Biosciences | Cat No. 562453 |
| PE-CF594 Rat anti-Mouse CD8a | BD Biosciences | Cat No. 562315 |
| PE-Cy5 Hamster anti-Mouse CD3e | BD Biosciences | Cat No. 553065 |
| PE-Cy5 Mouse anti-Mouse CD19 | Thermo Fisher | Cat No. 15-0193-81 |
| PE-Cy7 Mouse anti-Human CD73 | BD Biosciences | Cat No. 561258 |
| PerCP-Cy5.5 Hamster anti-Mouse CD27 | BD Biosciences | Cat No. 563603 |

**Supplementary Table 4. List of interactions between ligand and receptor molecular structures predicted by Molecular Operating Environment.**

| **Compound** | **Ligand** | **Receptor** | **Interaction** | **Energy (kcal/mol)** | **S-Score** |
| --- | --- | --- | --- | --- | --- |
| Luteolin (L) | OD1  CE1  6-ring | Asp 121  His 218  Phe 417 | H-donor  H-acceptor  Pi-Pi | -3.3  -0.8  -0.0 | -5.9896 |
| Quercetin (Q) | Cl  OD2  NE2  OD2  OD1  NE2  N01  OD2  NE2  OD2  OD1  OD2  ND1 | Cl 604  Asp 36  His 38  Asp 85  Asn 117  His 220  His 243  Asp 36  His 38  Asp 85  Asp 85  Asp 85  His 243 | H-donor  Metal  Metal  Metal  Metal  Metal  Metal  Ionic  Ionic  Ionic  Ionic  Ionic  Ionic | -2.0  -5.5  -5.9  -5.0  -3.8  -4.4  -6.5  -22.0  -20.6  -4.1  -3.4  -20.1  -18.4 | -6.5824 |
| Adenosine 5'-(α,β-methylene)diphosphate sodium (APCP) | OD1  OD2  NH1  NE2  NH1  NH2  ND2  ND2  NH1  NH2  ND2  Zn  Zn  NE2  NH1  NH2  Zn  NE2  NH1  NH2  NE2  Zn  NH2  NH1  NH2  NH1  NH2  OD2  NE2  OD2  OD1  NE2  ND1  OD2  NE2  OD2  OD1  OD2  ND1  6-ring | Asp 506  Asp 506  Arg 395  His 118  Arg 395  Arg 395  Asn 117  Asn 245  Arg 354  Arg 354  Asn 390  Zn 601  Zn 602  His 118  Arg 395  Arg 395  Zn 601  His 118  Arg 395  Arg 395  His 118  Zn 602  Arg 354  Arg 395  Arg 395  Arg 354  Arg 354  Asp 36  His 38  Asp 85  Asn 117  His 220  His 243  Asp 36  His 38  Asp 85  Asp 85  Asp 85  His 243  Phe 500 | H-donor  H-donor  H-acceptor  H-acceptor  H-acceptor  H-acceptor  H-acceptor  H-acceptor  H-acceptor  H-acceptor  H-acceptor  Metal  Metal  Ionic  Ionic  Ionic  Ionic  Ionic  Ionic  Ionic  Ionic  Ionic  Ionic  Ionic  Ionic  Ionic  Ionic  Metal  Metal  Metal  Metal  Metal  Metal  Ionic  Ionic  Ionic  Ionic  Ionic  Ionic  Pi-Pi | -3.1  -3.1  -10.6  -14.6  -2.6  -11.2  -1.3  -6.1  -9.3  -3.8  -1.6  -5.7  -5.6  -1.2  -5.5  -0.7  -23.9  -6.9  -3.5  -6.4  -1.5  -23.3  -1.9  -1.3  -1.8  -6.3  -2.3  -5.5  -5.9  -5.0  -3.8  -4.4  -6.5  -22.0  -20.6  -4.1  -3.4  -20.1  -18.2  -0.0 | -6.2695 |

**
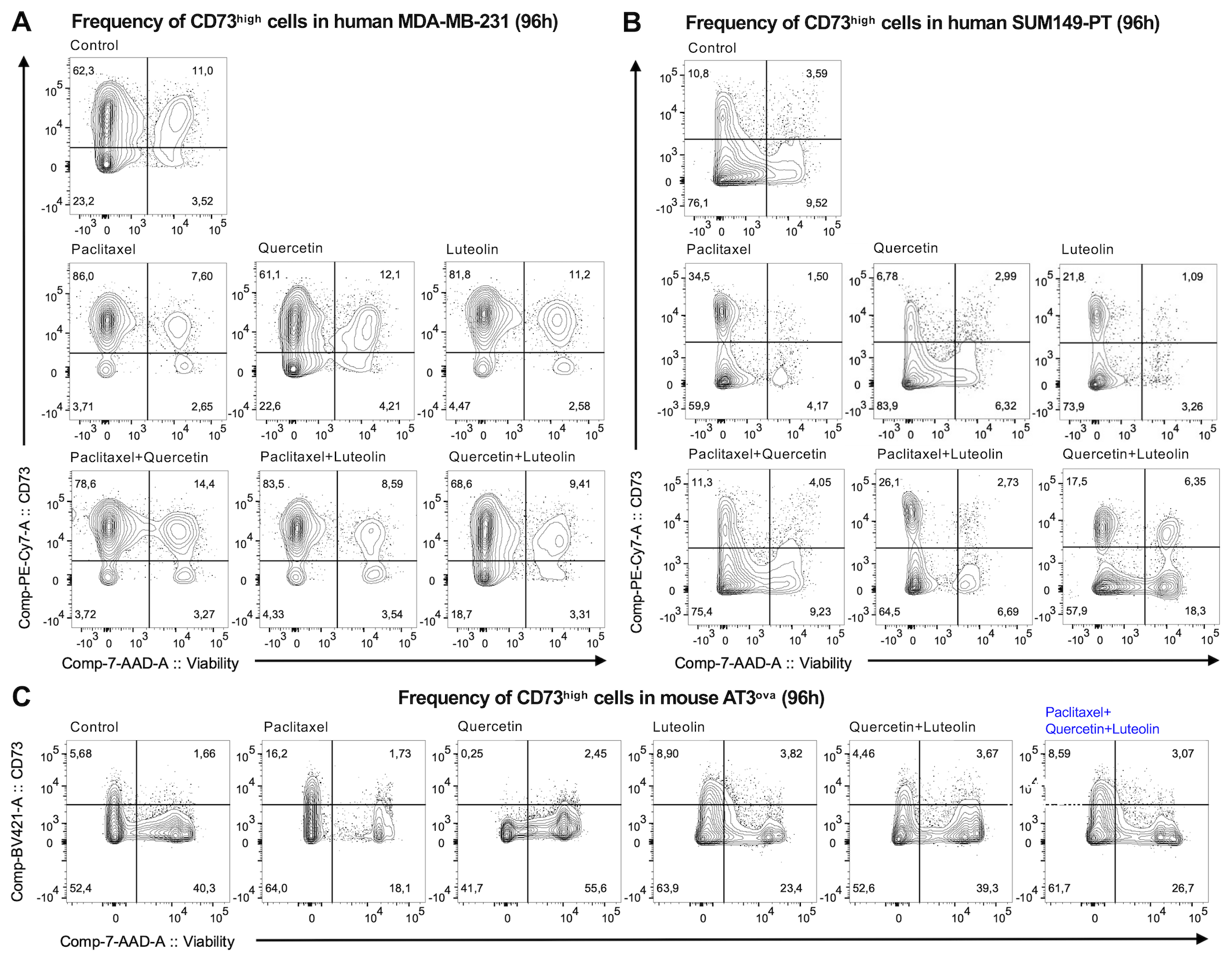
**

**Supplementary Figure S1. Natural compound quercetin effectively suppressed paclitaxel-mediated enrichment of CD73.** Representative cell surface expression of CD73 in human (**A**) MDA-MB-231 and (**B**) SUM149-PT and (**C**) mouse AT3^ova^ TNBC cell lines were assessed 96 hours post-treatment with paclitaxel (2.5 nM), quercetin (0.5 µM), luteolin (5 µM) or adenosine 5'-(α,β-methylene)diphosphate (APCP, 5 µM) alone and in different combinations. N=3, *, P<0.05; **, P<0.01; ***, P<0.005.

**
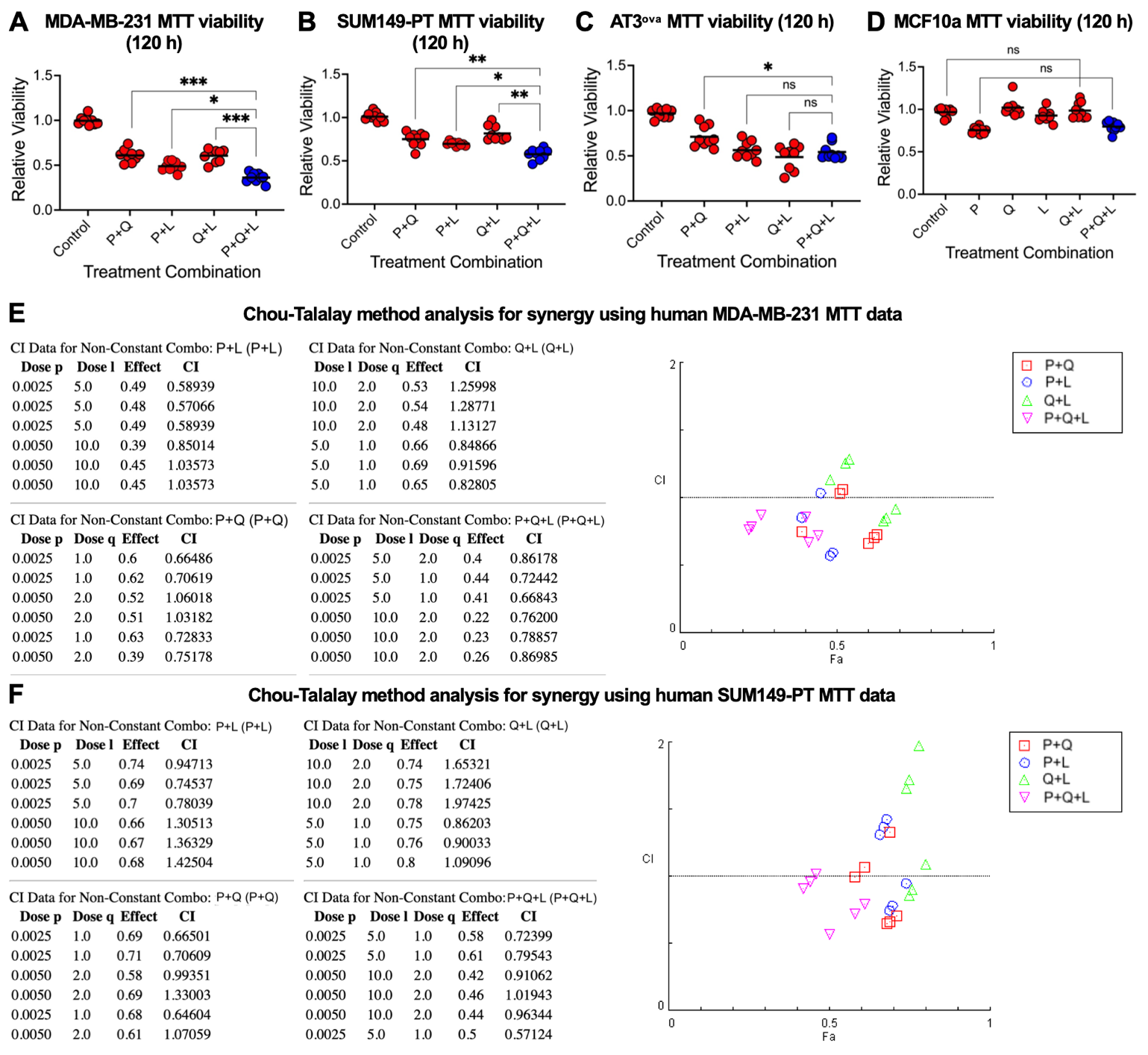
**

**Supplementary Figure S2. Triple-drug combination of paclitaxel, quercetin, and luteolin is the most effective drug combination.** Viability of (**A**) MDA-MB-231 and (**B**) SUM149-PT human TNBC, (**C**) AT3^ova^ mouse TNBC, and (**D**) MCF10a non-tumorigenic mammary cell lines were assessed in an MTT assay 120 hours post-treatment with paclitaxel (P, 2.5 nM), quercetin (Q, 0.5 µM), and luteolin (L, 5 µM) alone and in different combinations. Combination indices (CI) were determined for each possible drug combination using MTT cell viability data from (**E**) MDA-MB-231 and (**F**) SUM149-PT human TNBC cell lines, revealing the triple-drug combination to be synergistic. Data was inputted into CompuSyn software (PD Science LLC) to generate Chou-Talalay plots. CI>1, antagonism; CI=1, additive effect; CI<1, synergism. Data represents mean±SD, n=3, *, P<0.05; **, P<0.01; ***, P<0.001.

**
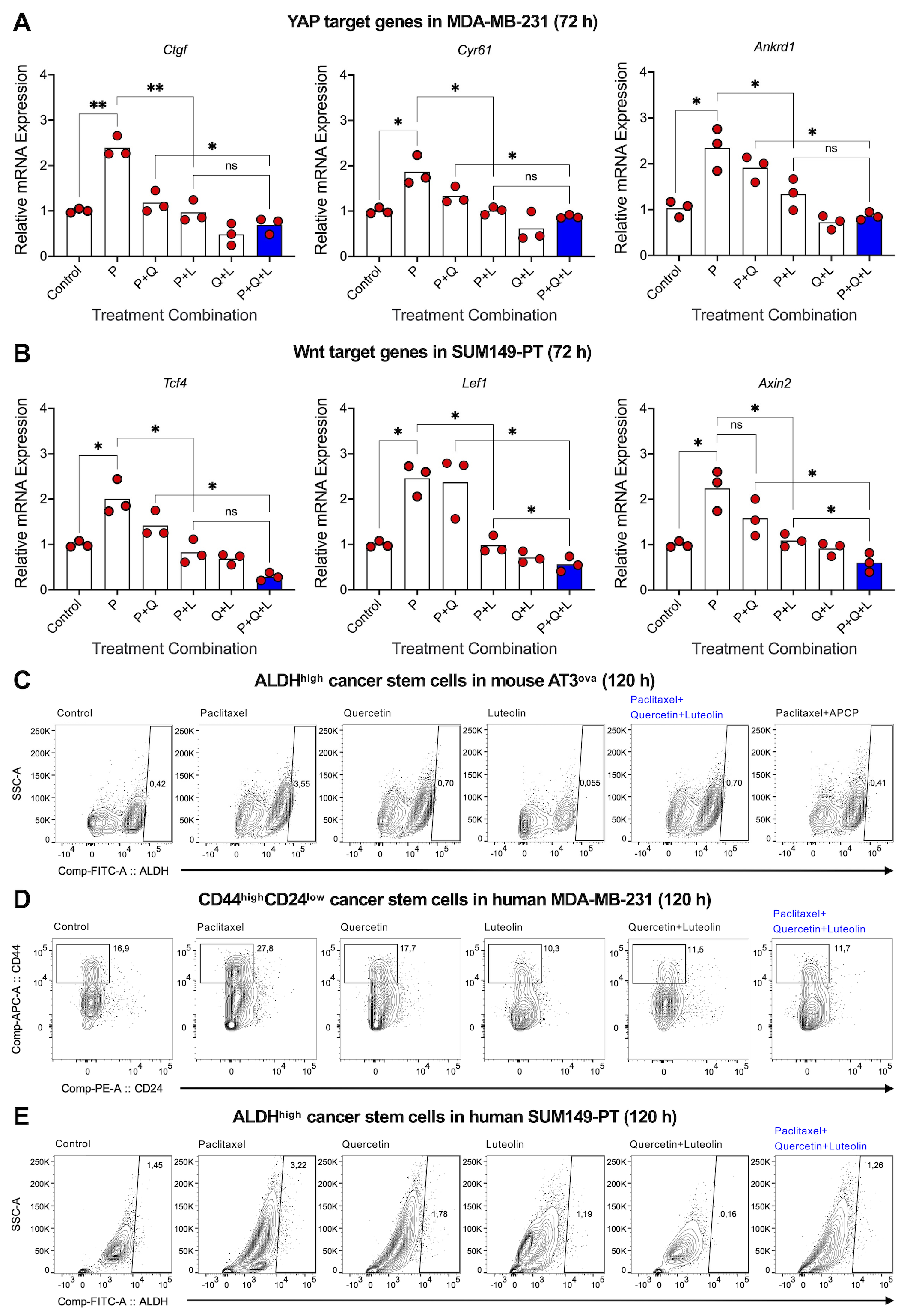
**

**Supplementary Figure S3. Natural compound luteolin effectively suppressed paclitaxel-mediated enrichment of cancer stem cells and their promoting pathways.** RT-qPCR analysis was carried out on (**A**) YAP target genes in MDA-MB-231 cells and (**B**) Wnt target genes in SUM149-PT cells 72 hours post-treatment with paclitaxel (5 nM), quercetin (1 µM), and luteolin (10 µM) alone and in different combinations. Representative frequencies of epithelial-like ALDH^high^ cancer stem cells in (**C**) mouse AT3^ova^ were observed 96 hours post-treatment with paclitaxel (2.5 nM), quercetin (0.5 µM), luteolin (5 µM) or adenosine 5'-(α,β-methylene)diphosphate sodium (APCP, 10 µM) alone and in different combinations. This was repeated on human (**D**) MDA-MB-231 and (**E**) SUM149-PT cells, revealing that luteolin antagonized paclitaxel-mediated enrichment on cancer stem cells more effectively than quercetin. Data represents mean±SD, n=3, *, P<0.05; **, P<0.01.

**
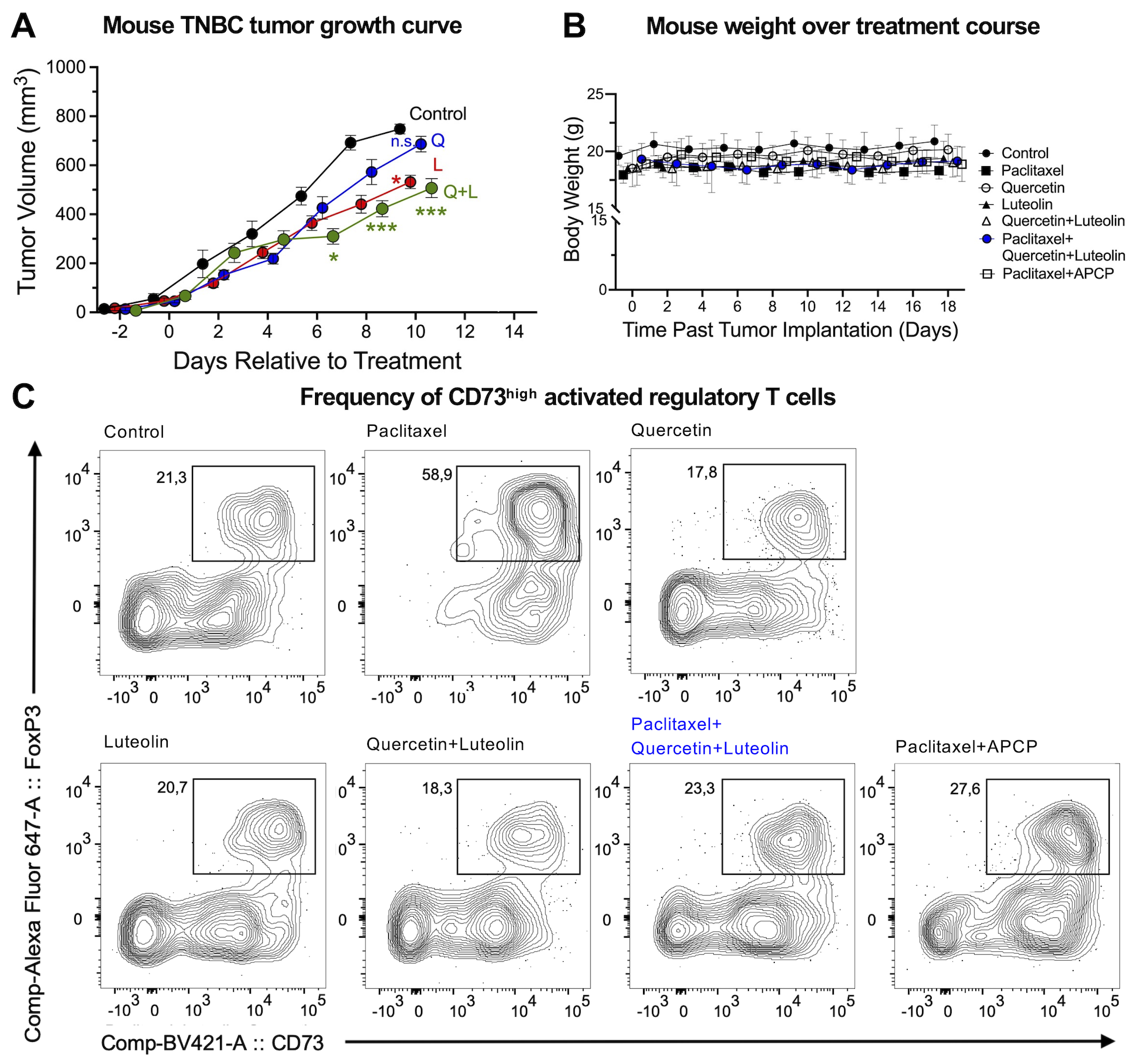
**

**Supplementary Figure S4. Triple-drug treatment was safe and reduced paclitaxel-enriched CD73^high^ activated regulatory T cells.** (**A**) Luteolin and quercetin reduced tumor growth of paclitaxel resistant AT3^ova^ mouse TNBC tumors both alone and in combination. (**B**) None of the treatments induced significant mouse body weight loss over the duration of tumor growth. Flow cytometric analysis of harvested tumors 12 days post-treatment revealed a decrease in the frequencies of (**C**) CD73^high^ activated regulatory T cells in response to triple-drug combination relative to chemotherapy alone. Data represents mean±SEM (A); mean±SD (B), n=6, *, P<0.05; **, P<0.005; ***, P<0.001.
